# PGT-A in POSEIDON Patients - perspectives and limitations

**DOI:** 10.5935/1518-0557.20250037

**Published:** 2025

**Authors:** Jakub Wyroba, Maria Barszcz, Iwona Fajt, Joanna Kochan

**Affiliations:** 1 Malopolski Institute of Fertility Diagnostics and Treatment - KrakOvi, Krakow, Poland; 2 Fertility Disorders Clinic, Andrzej Frycz Modrzewski Krakow University, Krakow, Poland; 3 Department of Animal Reproduction, Anathomy and Genomics, University of Agriculture in Krakow, Poland

**Keywords:** PGT-A, POSEIDON, ICSI

## Abstract

**Objective:**

The aim of this study was to determine the likelihood of being able to perform PGT-A, and its results, in each POSEIDON group compared to their age-matched non-POSEIDON group.

**Methods:**

This was a retrospective study of 4 groups of POSEIDON patients (n=824) who underwent intracytoplasmic sperm injection (ICSI) The controls were non-POSEIDON patients in two age groups (<35 and ≥35 years old; n=360).

**Results:**

The non-POSEIDON <35Y group had the highest number of embryos at the blastocyst stage that could be used for PGT-A (5.1±2/cycle), and the POSEIDON IV group had the fewest (0.6±0.3/cycle). Significantly fewer blastocysts were PGT-A tested in the groups with no indications for PGT-A (i.e. POSEIDON I (26%) and III (28%) and non-POSEIDON <35Y (39%)), compared to the groups with indications (i.e. POSEIDON II (69%), and IV (67%) and non POSEIDON ≥35Y (72%)). The euploidy rate was similar between groups without PGT-A indications (59%-64%) and between groups with indications (35-41%), but was significantly lower in groups with indications (*p*<0.001).

**Conclusions:**

POSEIDON patients are as willing to undergo PGT-A testing as non-POSEIDON patients, despite the poor prognosis. However, the final PGT-A result is very low compared to that in non-POSEIDON patients of the same age. Failure is usually caused by the inability to perform the blastocyst biopsy due to a lack of oocytes or blastocyst-stage embryos, and to a low rate of euploidy in groups ≥35Y.

## INTRODUCTION

**Figure 1:**
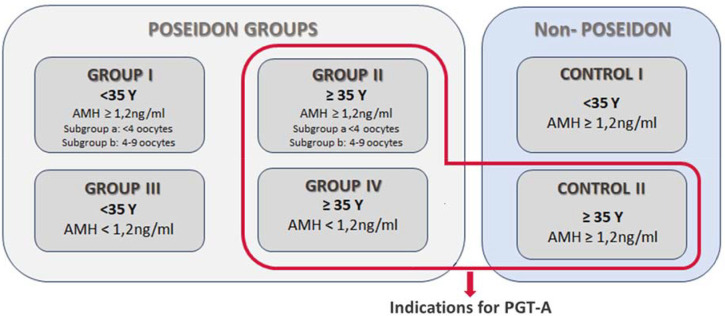
Classification of POSEIDON and non-POSEIDON groups.

One of the most difficult groups of IVF/ICSI patients to manage are those with a poor ovarian response (POR) to hormonal stimulation, which can occur in up to 30% of all assisted reproductive technology (ART) cycles (Jenkins *et al.*, 1991; Surrey & Schoolcraft, 2000; Giannelou *et al.*, 2020). Poor ovarian response is expected in patients with advanced maternal age and low AMH levels and they are mentally prepared for its occurrence. This is not the case, however, in patients with unexpected POR, i.e. young patients or those with normal AMH, for whom the low number of oocytes obtained after stimulation is surprising. For many years, the classification of POR was based on the Bologna criteria, which defined a poor response in IVF as the presence of at least two of the following three features: (a) advanced maternal age or any other risk factor for POR; (b) a previous POR; and (c) an abnormal ovarian reserve test (ORT) (Ferraretti *et al.*, 2011). The Poseidon Group *et al.* (2016) comprised of specialists in reproductive medicine and endocrinology, proposed a more detailed set of definitions for POR than those in the Bologna criteria, including the creation of 4 POSEIDON (Patient-Oriented Strategies Encompassing Individualized Oocyte Number) groups for low prognosis patients, based on age, AMH level, or oocyte number (Figure 1). Since then, a number of therapeutic paths have been proposed for specific groups of POSEIDON patients undergoing IVF (Humaidan *et al.*, 2016; 2019; Haahr *et al.*, 2019; Giannelou *et al.*, 2020; Sunkara *et al.*, 2020). Based on a multicenter cohort study, the cumulative delivery rate per IVF/ICSI cycle of is on average 50% lower in POSEIDON patients than in normal responders, and this varies across POSEIDON groups (Esteves *et al.*, 2021).

For over a decade, preimplantation genetic testing for aneuploidy (PGT-A) is a powerful tool in ART, offering the potential to enhance the chances of successful embryo implantation and pregnancy (Mastenbroek *et al.*, 2007; Yang *et al.*, 2022). This method is extremely important for patients of advanced maternal age, who have an increased risk of chromosomal abnormalities in their embryos (Franasiak *et al.*, 2014; Demko *et al.*, 2016; Conforti *et al.*, 2019; La Marca *et al.*, 2022). Therefore, indications for PGT-A are primarily in patients from POSEIDON groups II and IV (>35Y). However, PGT-A requires at least one good quality blastocyst on day 5 or 6 of culture, and we usually obtain relatively few oocytes, and just one or two embryos, in POR patients. If we maintain single embryos in culture until day 5, we risk embryo transfer (ET) failure due to the lack of a blastocyst, which causes the couple great stress, and can lead to their reluctance to undergo further IVF cycles. Therefore, there is a dilemma as to whether to offer POR patients PGT-A and risk a lack of blastocysts on day 5, or to transfer an untested embryo on day 3. The aim of this study was to determine the likelihood of being able to perform PGT-A, and its results, in each POSEIDON group compared to their age-matched non-POSEIDON group.

## MATERIALS AND METHODS

This was a retrospective study of 4 groups of POSEIDON patients (n=824) who underwent intracytoplasmic sperm injection (ICSI) in the Krakovi Clinic in Kraków (Poland) from 2021-2024. The controls were non-POSEIDON patients in two age groups (<35 and ≥35 years old; n=360). Figure 1 shows the criteria for including patients in the POSEIDON and control groups according to Poseidon Group *et al.* (2016). According to legal resolutions in our country, the indication for PGT-A is the maternal age ≥35 years old. The research was carried out in accordance with the guidelines of the local bioethics committee (KBKA/7/O/2024).

### Clinical protocols

Patients were treated using the long agonist protocol or short antagonist protocol. The type of protocol used depended on the level of AMH and the overall risk of hyperstimulation.

-Long agonist protocol: Starting 1 week before the expected menses (cycle day 18‒23), patients received the GnRH agonist, triptorelin (Decapeptyl, Ferring Pharmaceuticals, 1 mg/d, sc). After successful pituitary *downregulation* (when the serum estradiol (E2) levels were < 40 pg/mL), ovarian stimulation was commenced with a fixed daily dose of 150-300 IU recombinant follitropin alfa (rFSH, sc) with or without an additional 75‒150 IU menotropin (hMG).

-Antagonist protocol: A GnRH antagonist Cetrorelix (Cetrotide, Merck Europe, 0.25 mg/d, sc or Ganirelix, Gedeon Richter, 0.25 mg/d), was administered, commencing when the largest follicle reached a diameter of 14 mm. Administration of rFSH/hMG was initiated on cycle day 2-4.

The agonist and antagonist protocols were continued up to and including the day of human chorionic gonadotropin (hCG) administration, which was when the leading follicle reached a diameter of at least 18 mm and at least three follicles reached a diameter of at least 17 mm. rFSH was then stopped, and a single sc bolus of 10,000 IU hCG (Eutrig, Samarth Life Sciences) or 6,500 IU rhCG (Ovitrelle, Merck) was administered 36 h before the planned time of oocyte retrieval. When there was a risk of OHSS in an antagonist cycle, the trigger was a single sc bolus of triptorelin 2 mg, and a freeze-all policy was applied. All follicles 12 mm or larger were aspirated. Subsequently, the oocytes were inseminated via ICSI.

### Ovarian stimulation monitoring in ICSI

Baseline blood sampling and transvaginal sonography (TVS) were performed on day 2 or 3 of the treatment cycle for all patients. Monitoring of response during the treatment cycle consisted of TVS and blood sampling for hormonal analysis on cycle days 2-3 (E_2_, FSH, LH), 5-6 (E_2_), 8-9 (E_2_), and day of hCG administration (E_2_, P_4_). Additional TVS monitoring was performed as clinically indicated.

### Laboratory protocol

Oocyte-cumulus complexes (COCs) were identified using a stereoscopic microscope and then washed and incubated (approx. 3 h) in washing medium (Gynemed, Germany) under a 6.0% CO_2_, 5.0% O_2_ atmosphere. After incubation, oocytes were denuded using hyaluronidase and mechanical pipetting. Only oocytes in metaphase II (MII) with a first polar body were used for ICSI. For ICSI, fresh semen obtained by masturbation and analyzed according to WHO guidelines (WHO - World Health Organization, 2010) was used. The sperm sample was prepared for ICSI by density gradient centrifugation (Gynemed, Germany). Intracytoplasmic sperm injection (ICSI) was performed using an RI Integra 3 micromanipulator (Research Instruments, Germany) following the standard technique. Embryos were cultured in SAGE^®^ medium (Origio, Denmark) under an atmosphere of 6.0% CO_2_, 5.0% O_2_ and balance nitrogen at 37°C. Embryo development was assessed daily. Blastocysts were graded according to the Gardner scoring criteria (1). Blastocysts were biopsied using the same micromanipulator and microscope used for ICSI. The zona pellucida was perforated using an Octax® laser (Vitrolife, Sweden) for 250 μsec. The biopsied TE cells were washed with D-PBS and placed in 0.2 mL polymerase chain reaction (PCR) tubes for next-generation sequencing (NGS) by Igenomix Inc. (Spain).

### Statistical analysis

Non-parametric data, such as differences in the percentage values between groups, were assessed by the chi-squared test. Parametric data were expressed as means±SD and compared by two-way ANOVA. Differences were considered significant when the *p*-value was ≤0.05. The statistical analysis was performed using PQStat 1.6.2 (PQStat Soft, Poznan, Poland).

## RESULTS

**Figure 2:**
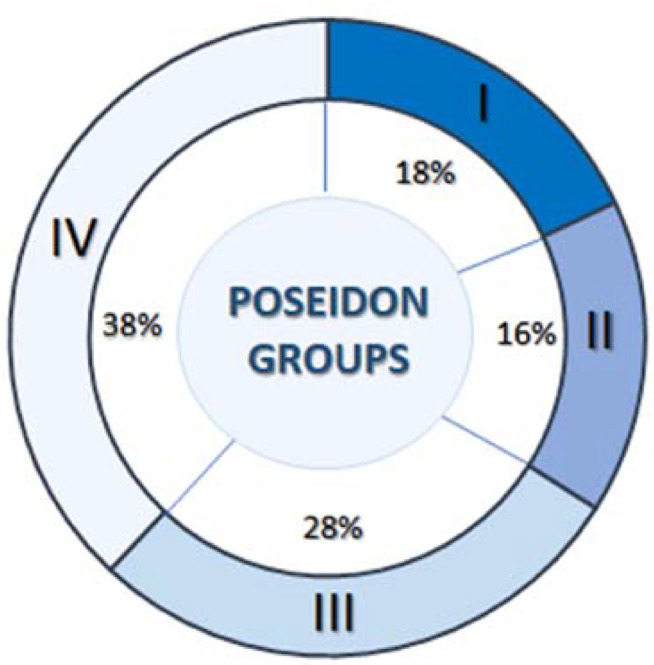
Patient distribution within POSEIDON groups.

A total of 824 cycles from POSEIDON patients and 360 cycles from non-POSEIDON patients were analysed. Figure 2 shows the distribution of cycles between the four POSEIDON groups. The largest group of POSEIDON cycles was group IV (n=314, 38%), while the smallest was group II (n=132, 16%). The basic characteristics of the patients and cycles are presented in Table 1. The lowest number of oocytes was obtained in POSEIDON groups III and IV (mean 2±1 oocytes/cycle), and these groups had the most cycles canceled due to lack of oocytes (16-19%). The highest number of oocytes was retrieved in Control group II (non-POSEIDON patients <35Y; mean 13±4).

**Table 1:** Baseline characteristics of the patients and cycles.

Parameters	POSEIDON				Non-POSEIDON		Total
	I	II	III	IV	<35 Y	≥35Y	
No of Patients n (%)	138	122	192	266	162	168	1048
No of cycles n (%)	148	132	230	314	176	184	1184
Age (years) mean±SD	32±3	38±2	31±3	38±2	33± 3	38± 3	34±5
BMI kg/m^2^ mean±SD	22±4	23±5	23±4	22±5	22±3	23±4	22±5
AMH ng/ml mean±SD	2.5±1.2^a^	1.8±0.7^b^	0.6±0.6^c^	0.7±0.5^c^	3.8±2^a^	2.9±0.9^a^	2.0±1
No of oocytes mean±SD	5±2^a^	4±1^a^	2±1^b^	2±1^b^	13±4^d^	10±3^d^	6±3
Cycle canceled due to lack of MII oocytes n (%)	10/148 (7%)^a^	12/132 (9%)^a^	34/230 (15%)^b^	56/314 (18%)^c^	0	0	
Cycle canceled due to lack of blastocysts (%)	19/148 (13%)^a^	20/132 (11%)^a^	34/230 (15%)^A^	53/314 (17%)^a^	12/176 (7%)^b^	18/184 (10%)^a^	

a:b, b:c, a:A, values with different superscripts within the same row differ significantly (<0.05).

a:c, a:d, b:d, A:b values with different superscripts within the same row differ highly significantly (*p*<0.001).

**Figure 3:**
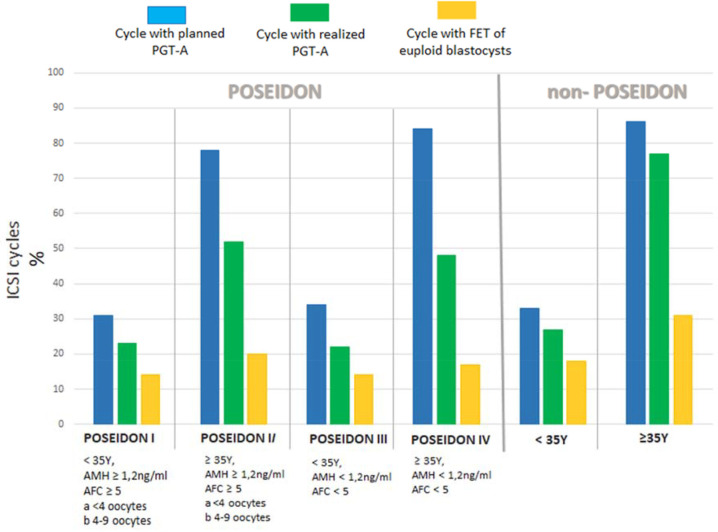
Planned and realized PGT-A test in POSEIDON and non-POSEIDON patients.

Table 2 shows the results of the PGT-A analysis in the POSEIDON and non-POSEIDON groups. The non-POSEIDON <35Y group had the highest number of embryos at the blastocyst stage that could be used for PGT-A (mean 5.2/cycle), and the POSEIDON IV group had the fewest (mean 0.6±0.3/cycle). Among the groups with indications for PGT-A, the non-POSEIDON ≥35Y group had the most blastocysts (mean 3.9±1) while the POSEIDON IV group had the fewest (mean 0.6±0.3/cycle). Significantly fewer blastocysts were PGT-A tested in the groups with no indications for PGT-A (i.e. POSEIDON I (26%) and III (28%) and non-POSEIDON <35Y (39%)), compared to the groups with indications (i.e. POSEIDON II (69%), and IV (67%) and non POSEIDON ≥35Y (72%)). Although a similar proportion of embryos were tested in groups with indications for PGT-A, this represented 0.4 and 0.7 blastocysts/cycle in the POSEIDON groups, and 2.8 blastocysts/cycle in the non-POSEIDON group (*p*<0.001; Figure 3). The euploidy rate was significantly higher in groups without PGT-A indications (59%-64%) compared to groups with indications (35-41%), (*p*<0.001).

**Table 2:** Results of the PGT-A test in POSEIDON and non-POSEIDON groups.

Parameters	POSEIDON				Non-POSEIDON		Total
	I	II	III	IV	<35 Y	≥35Y	
No of cycles n (%)	119	100	162	205	164	166	916
No of blastocysts	155	110	129	123	836	647	2000
No of blastocysts/cycle mean±SD	1.3±0.4a	1.1±0.3a	0.8±0.2a	0.6±0.3a	5.1±2c	3.9±1c	1.9±0.7
PGT-A tested blastocysts n (%)	40/119 (26%)^a^	80/110 (69%)^c^	36/129 (28%)^a^	82/123 (67%)^c^	326/836 (39%)^b^	456//647 (72%)^c^	1029/2000 (51%)
Euploid rate n (%)	24/40 (59%)^a^	30/80 (37%)^c^	22/36 (61%)^a^	29/82 (35%)^c^	151/326 (64%)^a^	187/456 (41%)^c^	504/1029 (49%)

a:b, b:c values with different superscripts within the same row differ significantly (*p*<0.05).

a:c values with different superscripts within the same row differ highly significantly (*p*<0.001).

## DISCUSSION

Couples undergoing IVF/ICSI are increasingly choosing preimplantation genetic testing of their embryos (PGT-A). This is related to increasing maternal age due to the postponement of procreation, which is associated with an increased risk of embryo aneuploidy and, consequently, the birth of an affected child (Franasiak *et al.*, 2014). Recently, it was proposed that having at least one euploid embryo for transfer would be a new marker of success in IVF/ICSI (Conforti *et al.*, 2019; Rodríguez-Varela *et al.*, 2025). This marker could be a good tool for clinicians when consulting patients and creating individual treatment plans.

Although several analyses have determined the chances of obtaining a euploid blastocyst in patients of different ages (Karlıkaya *et al.*, 2021) and with reduced ovarian reserve (Massie *et al.*, 2011; Luo *et al.*, 2022), these only considered IVF/PGT-A cycles. Here, we approached the question of preimplantation genetic diagnosis in POSEIDON patients holistically. In addition to estimating the euploid rate, we tried to determine the proportion of patients who decided to undergo PGT-A testing in the individual POSEIDON and non-POSEIDON groups, and how often the planned tests could be conducted. After consultation with a clinician and geneticist, approximately 30% of patients in the <35Y groups, and over 70% of patients in the ≥35Y groups, planned a PGT-A test. Patients aged >35Y with indications for PGT-A who decided not to undergo PGT-A were usually guided by economic or ethical considerations. POSEIDON patients over 35 years (groups II and IV) decided to undergo PGT-A testing as often as non-POSEIDON patients (Figure 2), even though there was a high probability that they would have their cycle canceled due to a lack of oocytes or embryos. In our study, 19% of patients from group IV and 9% from group II failed to obtain any MII oocytes. Another potential problem is the quantity and quality of embryos, with the need for at least one embryo at the blastocyst stage being a critical point in the IVF procedure that determining the possibility of performing PGT-A. In the POSEIDON cycles, we obtained only 0.6-1.4 blastocysts/cycle compared to 2.7-4.2 blastocysts/cycle in non-POSEIDON cycles (Figure 3), with the POSEIDON IV group, which has the most justified indications for PGT-A, having the fewest blastocysts available (0.6 blastocysts/cycle). Additionally, this group is the largest and constitutes 38% of all POSEIDON patients. This means that in the POSEIDON groups, PGT-A cannot be performed in 30-40% of cycles in which PGT-A was planned (Figure 2), while this is the case in only 10% of cycles in the non-POSEIDON >35Y group. Other authors report as well more than 30% cycle cancelled in poor prognosis patients, due to lack of oocytes or embryos (Klinkert *et al.*, 2004; Lamazou *et al.*, 2012; Berkkanoglu *et al.*, 2017).

It is well known that patients of advanced maternal age have an increased risk of aneuploidy (Awadalla *et al.*, 2021). This was reflected in our study, where the euploid rate of PGT-A tested blastocysts was 35-41% in the >35 Y groups and 59-64% the <35 Y groups. The slightly higher rate of euploidy in the non-POSEIDON groups is a reflection of not only having more blastocysts available, but also of being able to select the blastocysts with better morphology for PGT-A, whereas for the POSEIDON patients, all blastocysts underwent PGT-A, irrespective of their quality. The euploid rate we observed for patients >35 Y is consistent with that reported by other authors (Rubio *et al.*, 2017) and is not related to belonging to the POSEIDON groups.

Although the AMH level is not related to euploidy rates per embryo (Smeenk *et al.*, 2007; Massie *et al.*, 2011; Riggs *et al.*, 2011; Pipari *et al.*, 2021), it is indicative of low ovarian reserve and likely of collection a low numbers of oocytes and thus the number of embryos at the blastocyst stage. Therefore, patients from POSEIDON group IV, with low AMH and advanced maternal age ≥35Y, have the lowest chance of obtaining a blastocyst that can be used for PGT-A, as low AMH levels lead to a reduction in the number of oocytes and embryos. Further, their high maternal age leads to a high aneuploidy rate, and so these patients only manage to obtain an average of 0.14 euploid blastocysts/cycle, meaning that only every 10th patient in this group will complete their cycle with the transfer of a euploid blastocyst. Therefore, in this group, it would seem appropriate to consider an oocyte donation strategy, especially in patients of very advanced maternal age. Patients from POSEIDON group II are in a better situation, as although they are also of advanced maternal age, their higher AMH level makes it possible to obtain a larger number of oocytes, leading to the availability of more embryos for testing, and resulting in an average of twice as many euploid embryos per cycle (0.28) in comparison to group IV. In contrast, patients in the older control group (non-POSEIDON ≥35 Y) had an average of 1.5 euploid embryos/cycle. It should also be noted that in groups with indications for PGT-A, a similar proportion of patients decide to undergo PGT-A, regardless of the prognosis. If POSEIDON patients are unaware of the prospects and limitations of PGT-A testing, a poor outcome from their IVF/PGT-A cycle may come as a surprise, and cause a great psychological burden. Therefore, it is very important to thoroughly discuss the prospects and limitations of the PGT-A test with patients, especially those in groups with a poor prognosis. Low prognosis patients often have to decide, in consultation with their clinician and embryologist, whether to perform ET on day 3 without PGT-A or to leave a single embryo in culture until day 5, thereby risking the lack of a good-quality blastocyst for PGT-A.

A limitation of our research is that the POSEIDON groups are unequal. The smallest groups, I and II, are particularly problematic. We note that other authors have had a similar problem, with group II being the smallest (Eftekhar *et al.*, 2018; Chinta *et al.*, 2021). Due to the small number of patients in groups I and II, it was not possible to analyze live births after transfer of euploid embryos. The advantage of our research is that it was conducted in one center according to exactly the same protocols, which ensured the repeatability of the procedures.

In summary, POSEIDON patients are as willing to undergo PGT-A testing as non-POSEIDON patients, despite the poor prognosis. However, the final PGT-A result is very low compared to that in non-POSEIDON patients of the same age. Failure is usually caused by the inability to perform the blastocyst biopsy due to a lack of oocytes or blastocyst-stage embryos, and to a low rate of euploidy in groups >35Y.
